# Novel Biocompatible Polysaccharide-Based Eutectogels with Tunable Rheological, Thermal, and Mechanical Properties: The Role of Water

**DOI:** 10.3390/molecules25153314

**Published:** 2020-07-22

**Authors:** Huiping Xia, Meijie Ren, Yue Zou, Si Qin, Chaoxi Zeng

**Affiliations:** 1 Department of Food Science and Technology, College of Food Science and Technology, Hunan Agricultural University, Changsha 410128, China; hpxia@hunau.edu.cn (H.X.); 894653082@stu.hunau.edu.cn (M.R.); 201740717304@stu.hunau.edu.cn (Y.Z.); qinsiman@hunau.edu.cn (S.Q.); 2 Hunan Engineering Technology Research Center for Rapeseed Oil Nutrition Health and Deep Development, Changsha 410128, China

**Keywords:** natural deep eutectic solvents, xanthan gum, hydrogel, eutectogel

## Abstract

The natural deep eutectic solvent (NADES) is an excellent solvent for insoluble natural products and medicines. Eutectogels formed by gelation of NADESs are interesting materials that deserve attention. In this study, xanthan gum was used as a gelator to gel choline chloride-xylitol with different water contents in virtue of the excellent solubility of choline chloride-xylitol (1:1) to quercetin. We observed that water was critical to the formation of eutectogels. An MTT assay indicated that our eutectogel had excellent biocompatibility as its corresponding hydrogel. According to rheological tests, xanthan gum-based eutectogels had better viscoelastic properties, higher thermal stability, and more defined shear thinning behavior than its corresponding hydrogel. Texture profile analysis showed that eutectogels with less water content had higher hardness and adhesiveness. Meanwhile, Differential scanning calorimeter (DSC) results suggested that the various rheological and texture properties of eutectogels could be attributed to changes in the water state, which was influenced by the hydrogen bonding network of NADES. This biocompatible eutectogel with tunable properties was expected to find applications in novel drug delivery vehicles, which are widely used in the fields of medicine and food.

## 1. Introduction

Natural deep eutectic solvents (NADESs) are fabricated by mixing small organic molecules from various natural sources in specific molar ratios [1]. Due to its advantages of being natural, biodegradable, cheap, and of simple preparation, NADESs have attracted widespread attention in recent years as a new type of green solvent [2]. With the in-depth study on this new solvent, the applications of NADESs are expanding. Considering their environmental and safety characteristics, these solvents have great potential for applications in the food and pharmaceutical industries [3,4]. Among them, the strong dissolving properties of NADESs have attracted widespread attention and have proved to excel at dissolving many traditional poorly water-soluble or fat-soluble compounds [5,6].

Choi et al. measured the solubility of some natural products found in NADESs and found that the solubility of rutin in the solvent was 50 to 100 times higher than in water [7]. In addition, paclitaxel and ginkgolides, which are completely insoluble in water, also showed high solubility; the solubility in glucose-choline chloride reached 0.81 and 5.85 mg/mL, respectively. Biomacromolecules, such as DNA, albumin, and amylase, also showed good solubility in NADESs, and even the solubility of starch in glucose-choline chloride reached 17.2 mg/mL [7]. Dai et al. further determined the solubility of insoluble or sparingly-soluble natural products in NADESs. The solubility of these compounds was increased by 18 to 460,000 times compared to that in water [8]. Studies have shown that some flavonoid compounds display higher biological stability in NADESs than in traditional solvents [9]. Therefore, NADES is expected to serve as an excellent drug delivery vehicle for insoluble natural products and medicines.

In various drug delivery vehicles, hydrogels have received considerable attention because they are closer to living tissues than other synthetic materials. The use of hydrogels has extended to various biomedical and pharmaceutical applications, making use of oral, transdermal, and other routes of administration [10]. Recently, eutectogels have been fabricated via gelation of deep eutectic solvents using a chemically-synthesized gelator; therefore, it is also possible to form gels based on NADESs [11,12]. It has been suggested that NADESs could be the liquid, other than water and oil, found in biological cells [7,13]. Studies have also found that the cytoplasmic composition of cells resembles a gel-like formation of various biological small- and macromolecules [14]. Therefore, NADESs-based gels could represent a better drug delivery vehicle than hydrogels for poorly soluble bioactive ingredients and medicines.

Xanthan gum is an anionic microbial polysaccharide produced commercially via bacterial fermentation. As a versatile biopolymer, xanthan gum has been widely used in various industrial and biomedical applications, such as food, cosmetics, and drug delivery [15]. Xanthan gum has recently attracted significant attention as a biomaterial for the preparation of tissue scaffolds (extracellular matrix) for tissue engineering applications [16]. To the best of our knowledge, there are no studies on the preparation of eutectogels using xanthan gum as a gelling agent for potential applications as drug delivery vehicles. In this study, we prepared and evaluated the biocompatibility of biodegradable xanthan gum-based eutectogels based on NADES choline chloride-xylitol (ChCl-Xyl, 1:1), which is highly soluble to quercetin. The influence of water content on the properties of the eutectogel and its possible mechanism were analyzed. Our results were expected to guide the application of this novel gel in various fields, such as medicine and food.

## 2. Results and Discussion

### 2.1. Preparation and Biocompatibility Evaluation of Xanthan Gum-Based Eutectogels

Quercetin is a typical flavonoid with antioxidant, anti-inflammatory, and antibacterial activities; for these reasons, it has important applications for human health [17]. However, flavonoids, such as quercetin, often have the disadvantage of extremely low water solubility. Studies have explored various delivery vehicles to improve their bioavailability [18,19]. NADES is considered an ideal drug delivery vehicle, which is also proven here for quercetin. Figure S1 shows the solubility of quercetin in choline chloride-xylitol (1:1, ChCl-Xyl) with different water contents. It was found that the solubility of quercetin in ChCl-Xyl with 20% water content increased by more than 10,000 times compared to that in water. The lower the water content in the solvent, the greater the solubility of quercetin. ChCl-Xyl was thus an ideal quercetin delivery vehicle. For this reason, we chose ChCl-Xyl as a typical NADES to investigate its gel preparation process and the effect of water content on its properties.

**Figure 1:**
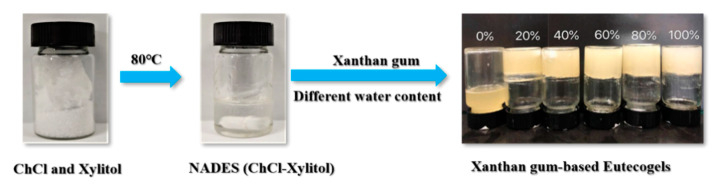
The preparation process of xanthan gum-based eutectogel.

Figure 1 shows the preparation process of ChCl-Xyl eutectogels based on xanthan gum. The preparation process of the NADES ChCl-Xyl is simple and convenient, with only two natural components required to be mixed at specific molar ratios at 80 °C. NADES is a green solvent with a broad potential for applications. When xanthan gum was added into the ChCl-Xyl under anhydrous conditions, the gel did not form even with sufficient stirring. However, stable gels were formed upon the addition of water and after annealing treatment. It is generally accepted that the formation of polysaccharide gels occurs mainly due to the rearrangement of the polymer molecular structure in the solvent [20]. For example, the formation of a xanthan gum hydrogel is described as the reconstruction of xanthan molecule network structure into a more ordered network structure under annealing conditions with the help of non-freezing water [21]. Therefore, we speculated that in the dissolution of ChCl-Xyl, the solvent could not completely play the role of non-freezing water to promote the rearrangement of xanthan polymer, and the presence of water played a key role in the formation of the eutectogels.

**Figure 2:**
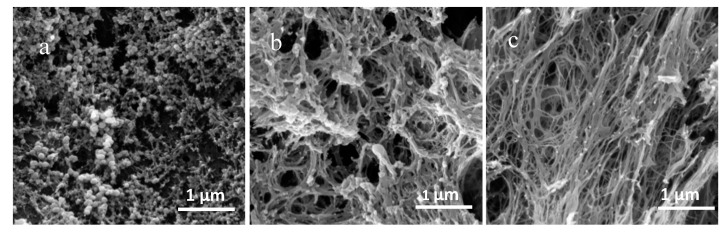
The SEM images of xanthan gum-based hydrogel (**a**) and eutectogels with different water contents (**b**) 60%, (**c**) 20%.

Figure 2 shows the SEM images of eutectogels with different water contents (20% and 60%) and xanthan gum-based hydrogel. It was found that different gel microstructures were found among these xanthan gum-based gels. Because the choline chloride-xylitol had been removed from the eutectogels, the SEM images showed the microstructure of the xanthan gum in the gels. It could be seen that different water contents had a significant effect on the structural reconstruction of xanthan gum during the formation of eutectogel.

**Figure 3:**
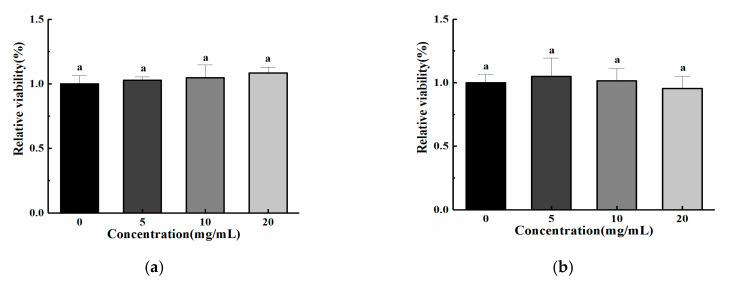
Detection of toxicity of xanthan gum hydrogel (**a**) and xanthan gum eutectogels with 20% water content (**b**) by MTT assay. “a” is xanthan gum hydrogel.

An MTT assay was used to quantitatively assess the biocompatibility of ChCl-Xyl eutectogels. After 24 h of incubation, the cell activity in all concentrations of eutectogel (20 wt% water content) and hydrogel extract was around 100% compared to the control group using normal medium (Figure 3). There was no significant difference in cell activity between the experimental group and the control group (*p* > 0.05). Therefore, our results indicated that these eutectogels had excellent biocompatibility as their corresponding hydrogel.

### 2.2. The Gel Properties of Eutectogels with Different Water Content

#### 2.2.1. Rheological Properties

**Figure 4:**
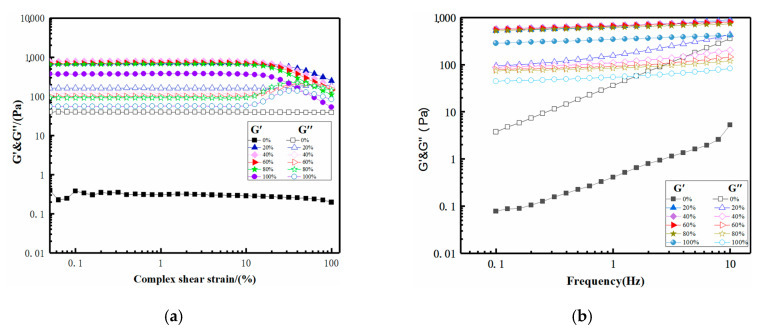
Strain sweep (**a**) and frequency sweep (**b**) curves of the xanthan gum-based eutectogels with different water contents and hydrogel.

Figure 4a shows the strain sweeps of eutectogels with different water contents and hydrogel. The elastic modulus (G′) and viscosity modulus (G″) were used as response values to investigate the variation of the modulus of different gel samples with strain amplitude. Except for the anhydrous NADES-based vehicle, the G′ values of the other five samples with different water contents in the linear viscoelastic region (LVR) were greater than the G″ values, indicating that gels were formed. This could also be proved from the low dynamic loss tangents (tan δ = G″/G′, tan δ < 1 indicated predominantly elastic behavior) values of eutectogels with different water contents (Table 1). Meanwhile, with increasing water content, the LVR narrowed gradually. This suggested that adding more water to the vehicles made the eutectogels more brittle and less resistant to structural degradation.

Compared with hydrogels, the eutectogels have a larger G′LVR and a longer LVR. Longer LVR causes eutectogels with less water to exhibit greater elasticity and toughness to resist deformation. The difference in viscoelastic properties between the eutectogels and hydrogel may be attributed to the different conformation of xanthan gum in the solvent [22]. Carnali et al. proposed that the linear viscoelastic behavior in concentrated xanthan gum solution might be attributed to the formation of weak three-dimensional networks and complex aggregation in solution through physical entanglement and hydrogen bonding between xanthan gum molecules [23]. Song et al. proposed that the strain-thinning behavior of the G′ of concentrated xanthan gum solution under large strain amplitudes could be explained by the destruction and formation of its internal structure in response to external stimuli [24]. Hence, the larger G′LVR values and longer LVR may be attributed to more ordered three-dimensional networks of xanthan gum molecules in the eutectogels.

Frequency sweep was performed to study the dependence of the material on the deformation rate. Figure 4b shows the frequency correlation of G′ and G″ of xanthan gum-based eutectogels with different water contents and xanthan gum-based hydrogel under constant strain in LVR. Samples were classified into strong gels, weak gels, and viscous sols based on their frequency sweep. When the elastic modulus G′ was plotted as a function of frequency, strong gels showed a constant value (frequency independence), while weak gels showed a frequency-dependent behavior (G′ increased with increasing frequency). Although we observed slightly positive slopes of G′ curves, which were characteristic of weak gels, the G′ values of the eutectogels had an almost independent frequency response, evidencing that the eutectogels had a good tolerance to the applied rate of deformation. Meanwhile, it was widely accepted that the G″ values increased proportionately to the scan frequency, indicating that the internal network of eutectogels was composed of non-covalent interactions. Figure 4b shows that, with increasing water content, the G″ values of the eutectogels showed a more independent frequency response, suggesting less non-covalent interactions.

**Figure 5:**
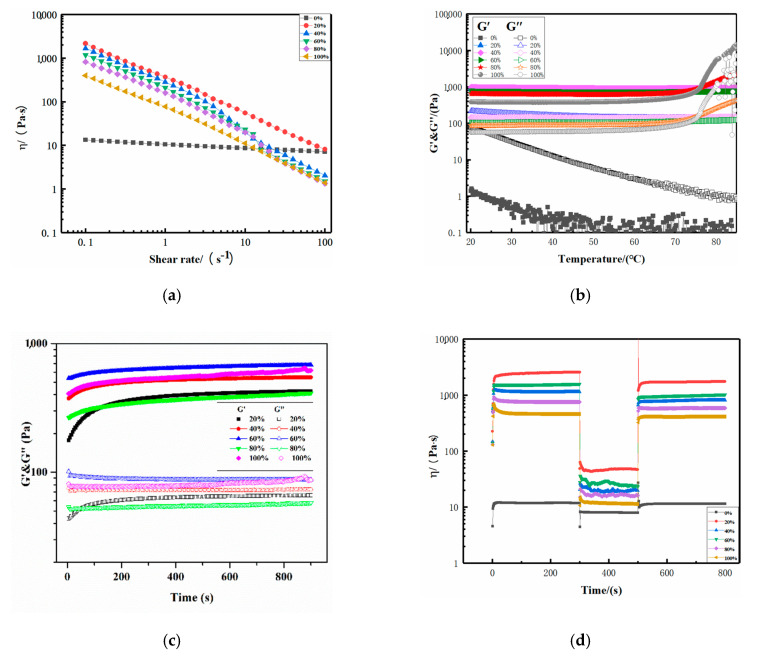
Flow measurement (**a**), temperature sweep (**b**), time sweep (**c**), and recovery tests (**d**) results of the xanthan gum-based eutectogels with different water contents and hydrogel.

The flow measurements of the gel samples were extracted from the apparent viscosity of the sample with the shear rate. Figure 5a shows that, under low shear rate, the viscosity of eutectogels decreased with increasing the water content. This might be primarily due to the reduced viscosity of the diluted NADESs, as the addition of water to the NADES has been reported to significantly reduce the viscosity of the solvent [25]. Meanwhile, all the eutectogels exhibited a pseudoplastic behavior, with the apparent viscosity decreasing with increasing the shear rate. As with hydrogels, the relationship between apparent viscosity and a shear rate of eutectogels with 20% water content conformed to the power-law model. Interestingly, eutectogels with 40, 60, and 80% of water content showed distinct trends. Under the high shear rate and with the increase of water content, the viscosity of eutectogels decreased more rapidly, which was closer to the behavior of hydrogels. This unique property was beneficial for the biodegradable eutectogels as it met the standards of biomedical materials, such as injectable gels.

Figure 5b shows the temperature sweep curves of gel samples with different water contents. The G′ values of all water-containing samples were higher than their G′ values, and no gel-sol conversion occurred, meaning that all eutectogels and hydrogels remained “gelatinous” at high temperatures. We confirmed that, as reported in the literature, xanthan gum-based gels showed good thermal stability [26]. For hydrogels, the G′ and G″ values increased significantly after the temperature reached 76 °C. The eutectogel with 80% water content also showed increased G′ and G″ values. However, other eutectogels kept their modulus values constant throughout the whole temperature range. This indicated that the eutectogels had better thermal stability than the hydrogels. A time sweep experiment was used to investigate the gelation process of eutectogels. As shown in Figure 5c, the eutectogels with different water contents, like their corresponding hydrogel, had a rapid increase in G′ value within two minutes at 80 °C incubation, and then the growth rate slowed down significantly. This showed that eutectogels with different water contents had the characteristics of rapid gelation.

We studied the time-dependent fluid behavior to gain insights into the thixotropy and structure-recovery properties of these xanthan gum-based eutectogels and hydrogels. The structure recovery properties were evaluated by using the three-interval time test (3ITT). In this test, the viscosity changes were followed as a function of time under alternating constant shear rates (0.1, 10, and 0.1 s^−1^). The structure recovery was calculated for each sample by taking the viscosity value at the end of interval 1 as 100% and comparing it with the peak viscosity value in interval 3. The percentage of recovery for all samples exceeded 80%, indicating a good structural recovery (Figure 5d). Meanwhile, it was observed that, as the water content of the eutectogels increased, the structural recovery of the eutectogels also increased proportionally.

#### 2.2.2. DSC Measurements

Bound water plays a key role in the formation of xanthan gum-based hydrogels, with more bound water being beneficial to the formation of a more ordered and stable conformation of xanthan gum [27,28]. Therefore, the various gel properties of eutectogels with different water content may be related to the different states of water.

DSC is an effective method to determine the state of water in gels. It has been reported that the melting and crystallization behavior of water changes with the structure of gels. Therefore, the melting and crystallization peaks of water in the gel can be used as an indicator of the structural change of the gel [29]. We used DSC to further investigate the influence of different water contents on the structure and properties of the xanthan gum-based eutectogels. Generally, water in polysaccharide-based gels can be divided into “free water”, “freezing bound water”, and “non-freezing bound water” [28]. The properties of free water in the hydrogel are considered the same as pure water because it is not affected by polysaccharide molecules. However, although it crystallizes as free water, the freezing bound water has a lower melting temperature due to the relatively weak hydrogen bonding interaction between freezing bound water and polysaccharide molecules. Non-freezing bound water cannot crystallize, and it is tightly bound to the polysaccharide molecular chain by hydrogen bonding. Takahashi et al. found that non-freezing water molecules played an important role in the gelation process of xanthan gum gels, and the structural changes of xanthan gum/water vehicle were characterized by the desorption and adsorption behavior of non-freezing water [30].

**Figure 6:**
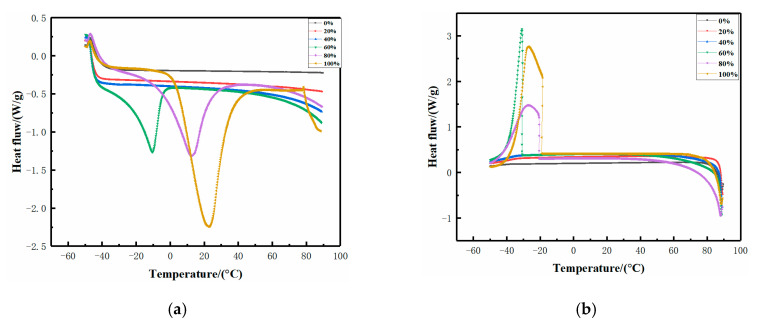
DSC heating curves for xanthan gum-based eutectogels with different water contents and hydrogel (**a**); DSC cooling curves for xanthan gum-based eutectogels with different water contents and hydrogel (**b**).

Figure 6a shows the DSC heating curves of eutectogels with different water contents. Eutectogels with 20 and 40% water content did not show an endothermic peak within the range of temperatures tested. It is well known that there are no detectable phase transitions in non-freezing bound water in the temperature range of −73 to 0 °C. This indicated that the water molecules in these two eutectogels were forming non-freezing water. The area under the DSC peak in the heating curve represented the enthalpy change associated with the melting of freezing water (free water and freezing bound water). There were wide absorption peaks in the gels with 60, 80, and 100% water content, and this was because of the melting of freezing water in the gels. Water with melting temperature (Tm) at 0 °C was free water, while water with melting temperature Tm below 0 °C was freezing bound water. Only a single exothermic peak appeared in these gels, and the Tm values were all lower than 0 °C. Thus, the eutectogels with 60 and 80% water content and hydrogels might contain both freezing bound water and free water. This was verified in the DSC cooling curve (Figure 6b), which showed that evident single exothermic peaks appeared only in gels with water contents of 60, 80, and 100%.

With increasing water content, the exothermic peak moved to higher temperatures, meaning that the proportion of free water increased. However, the peak areas of DSC in eutectogels with 60 and 80% of water content were similar. The gel with the highest water content did not show a larger endothermic peak area, indicating that bound water still accounted for the majority of water, which was expected in virtue of the nature of NADESs. Based on the supramolecular hydrogen bond network composed of NADESs, water could be regarded as one of the parts of the solvent and participate in the construction of supramolecular hydrogen-bond networks. Therefore, compared with the hydrogels, the structure of water in eutectogels was predominantly non-freezing water and freezing bound water. Higher bound water content indicated that xanthan gum molecules had formed a more ordered and compact structure composed of hydrogen bond interactions after annealing treatment, which also explained why the eutectogels had better gel properties than hydrogels.

#### 2.2.3. Texture Profile Analyses

**Figure 7:**
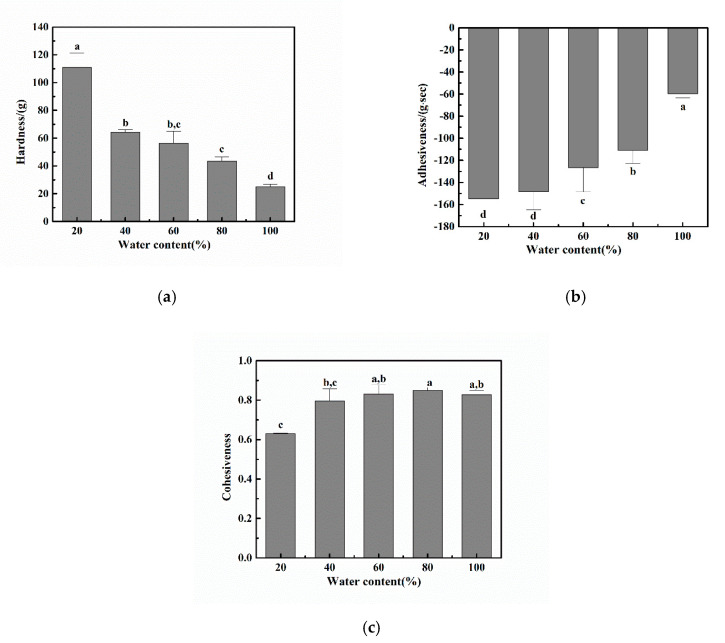
The mechanical properties of eutectogels with different water contents and hydrogel. (**a**) Hardness, (**b**) adhesiveness, and (**c**) cohesiveness (*n* = 6). Bars indicate standard deviation (S.D.). Statistical differences withing each mechanical property are denoted by the small letters a–d, values presenting the same small letters in the figure mean that the samples had no significant differences between different water content.

Texture profile analyses (TPA) provided further information on the gel properties of eutectogels. Figure 7 shows the effect of different water contents on the texture properties of four eutectogels and the hydrogel. Several mechanical parameters were determined from the force-time diagram through TPA analysis, including hardness, adhesiveness, and cohesiveness. Hardness is the force required to produce gel deformation. The hardness of the eutectogel with 20% water content was 112.5 g (± 12.4), which indicated that the eutectogels with lower water contents had a higher resistance to deformation than the hydrogel (Figure 7a). As the water content increased, the hardness of the eutectogels decreased sharply. It is generally accepted that the resistance of the hydrogel to deformation is related to the degree of physical cross-linking of the gelator inside the gels. Therefore, xanthan gum-based eutectogels with lower water displayed a more ordered conformation of the xanthan gum and consequently displayed higher hardness values too.

Adhesiveness relates to the ability of a material to adhere to a given surface and is an important indicator of the quality of a hydrogel, especially in the preparation of adhesive drug delivery vehicles. Adhesiveness is a surface property arising from the combined effects of adhesion, cohesion, viscosity, and viscoelasticity [31]. It is defined as the negative force area of the first compression cycle and represents the work required to overcome the attractive force between the gel surface and the probe surface. Figure 7b shows that, with increasing the water content of the eutectogels, the adhesiveness of the samples gradually decreased. The adhesiveness of the xanthan gum hydrogel was the lowest. We found that the xanthan gum-based eutectogels had better adhesiveness than their corresponding hydrogel.

The stability of the internal structure of gels is related to cohesiveness. Cohesiveness is defined as the ratio between the area under the force-time curve produced during the second compression cycle and the area produced during the first compression cycle. In other words, cohesiveness is the bearing capacity of a sample relative to its behavior under the first deformation and measures how well the sample maintains its structure after the first compression. This value indicates the ability of gels to partially maintain its three-dimensional structure when subjected to compressive stress. The cohesiveness of eutectogels with different water contents was in the range of 0.6 to 0.9, and there was no significant difference in the cohesiveness of the eutectogels with water contents above 40%. Therefore, all the gels showed a good ability to maintain their structural integrity (Figure 7c).

## 3. Materials and Methods

### 3.1. Materials

Commercially available xanthan gum from Xanthomonas campestris (C35H49O29, Mw = 600 kDa, USP grade, 95–99%, xanthan powders contain moisture of ca. 12% and ash of 5.5–13% cps) and xylitol (purity 98%) were purchased from Aladdin Ltd. (Shanghai, China). Choline chloride was purchased from Sinopharm Chemical Reagent Co., Ltd. (Beijing, China) and used as received. Human hepatoma G2 (HepG2) cell line was purchased from the Shanghai Institute of Cell Biology. MTT [3-(4,5-dimethylthiazol-2-yl)-2,5-diphenyltetrazolium bromide] and DMSO were purchased from Solarbio Science and Technology Co., Ltd. (Beijing, China).

### 3.2. NADES and NADES-Based Eutectogels Preparation

NADES were prepared according to the procedure described in our previous work [32]. Choline chloride-xylitol (ChCl-Xyl, 1:1) was prepared by mixing choline chloride and xylitol at a molar ratio of 1:1. The resulting mixture was placed in a screw-capped vial with a stirring bar. Then, the mixture was heated with agitation at 80 °C for more than 30 min until a clear liquid was obtained.

Xanthan gum-based eutectogel preparation: The appropriate amount of NADES and xanthan gum (5% of the total mass of the gel) in screw cap vials was accurately weighed, stirred, and mixed at room temperature while adding different amounts of deionized water (0, 20, 40, 60, and 80% of the total mass of the gel). Then, the sample vials were heated with stirring in a water bath at 80 °C for 5 min. Eutectogels were formed by cooling vials at room temperature. The preparation of xanthan gum-based hydrogel was the same as that of the eutectogel.

### 3.3. Solubility Measurement

The solubility of quercetin in ChCl-Xyl with different water contents was measured using the shake flask method [33]. An excess quantity of quercetin was added to each vial containing approximately 20 mL of ChCl-Xyl and stirred at 25 °C for several hours. The supersaturated solution was centrifuged at 10,000 g for 5 min and then filtered using a 0.45 μm Polyethersulfone (PES) syringe filter. At least 3 independent analyses were performed to obtain each result. If necessary, the mixtures were diluted with ethanol, after which, the concentration of quercetin in water and choline chloride-xylitol with different water contents was determined, using a UV-vis spectrophotometer (model TU-1901, Beijing, China) at 372 nm. A standard curve (y = 0.01189 + 63.42807x, R^2^ = 0.9952) was constructed to quantify the mass fraction of dissolved quercetin.

### 3.4. Morphology Characterization

SEM images were acquired using an FEI Quanta FEG 250 scanning electron microscopy (SEM) (FEI Corporate, Hillsboro, OR, USA) with an operating voltage of 30 kV. Samples were prepared by suspending the eutectogels in ethanol vial for 5 days, and the anhydrous ethanol was changed every 24 h until the NADES was completely replaced. The soaked samples were quickly frozen and converted to xerogels by freeze-drying for several hours. The samples were then introduced into the SEM machine and sputter-coated with a thin film of gold prior to imaging.

### 3.5. Biocompatibility Evaluation

The gel samples were accurately weighed and mixed with DMEM (high glucose) medium containing 10% (*v*/*v*) fetal bovine serum in a proportion of weight:medium volume of 1:10 and placed in an incubator at 37 °C for 24 h. The obtained extract was filtered using a 0.22 μm filter and further sterilized. The concentration of the extract was 100 mg/mL. Normal cultured cells were used as the control group, and the corresponding gel samples were added to the sample group. Each sample was diluted with medium to 5, 10, and 20 mg/mL, respectively. HepG2 cells were seeded in 96-well plates (4 × 10^3^ cells/well) in a volume of 100 mL of medium and were allowed to attach for 24 h before treatment. After culturing for 24 h, the medium containing the sample was removed. Cell cultures were washed three times with PBS buffer solution, and 100 μL of medium containing 0.5 mg/mL MTT was added, followed by 4 h incubation at 37 °C and 5% CO_2_. After discarding the medium, 100 μL of DMSO was added to each well, and the absorbance at 570 nm was measured using a microplate reader (Multiskan Ascent, Thermo Fisher Scientific, Waltham, MA, USA). Relative vitality % = OD value of experimental group/OD value of control group × 100.

### 3.6. Rheological Measurements

The rheological measurements were conducted in a Kinexus Pro rotational rheometer (Malvern Instruments, Worcestershire, UK) equipped with a stainless-steel cone. A parallel plate geometry cell (40 mm diameter) with a 1 mm gap between plates was used. Typical rheology experiments used 1 Hz as the initial value to test the gels in an amplitude sweep. A sweep of strain from 0.01 to 100% was used for the tests. The frequency sweep measured shear storage modulus (G′) and loss modulus (G″) as a function of frequency over the range of 0.1 to 10 Hz. To determine the viscosity, dynamic flow sweep was investigated with a shear rate from 0.1 to 100 s^−^^1^. The time scan was used to investigate the time dependence of the G′ and G″ of the gelation process at 80 °C. The strain and angular frequency were set to 1% and 1 Hz, respectively. A temperature sweep with a heating rate of 1 °C/min at a constant frequency of 1 Hz and a fixed strain of 1% was carried out from 20 to 90 °C. All the experiments were conducted at 25 °C, and all samples were equilibrated for 2 min before measurements.

### 3.7. Texture Profile Analyses

Texture profile analysis (TPA) of the eutectogels and hydrogel in TPA mode was performed using a TA-XT plus texture analyzer (Stable Micro Systems, Surrey, UK). The samples were compressed twice (depth 10 mm; delay time 5 s) under a cylindrical probe (P/0.5) with a controlled force of 5 g. The pre-speed, operating speed, and post-speed were 2, 1, and 2 mm/s, respectively. Force-time curves were generated by Texture Exponent Lite software during two cycles of compression, from which hardness, cohesiveness, and adhesiveness were determined.

### 3.8. DSC Measurements

To assess the state of water in the xanthan gum-based eutectogels with different water contents and hydrogel, calorimetric experiments were performed using a DSC instrument (Q2000 DSC, TA instruments, New Castle, DE, USA) operating in the Heat Flow T4P modality. Measurements were performed under dry high-purity helium at a flow rate of 50 mL/min. Roughly 10 mg of the samples was weighted in a standard Tzero aluminum pan. The temperature was raised from room temperature to 90 °C. Then, the temperature was reduced to −50 °C at a rate of 10 °C/min. Finally, samples were heated up to 90 °C at a rate of 10 °C/min. From this information, the thermal curve and related thermal parameters during melting and crystallization were extracted.

### 3.9. Statistical Analysis

All measurements were repeated at least three times using duplicate samples. The mean and standard deviations were calculated from these data. The statistical analyses involved analysis of variance (ANOVA) using Minitab 16 (Minitab Inc., State College, PA, USA) with Tukey’s test with a *p* < 0.05 considered as statistically significant.

## 4. Conclusions

As a new type of green solvent, NADES has received significant attention in recent years due to its excellent solubility for bioactive ingredients. Eutectogels based on natural polysaccharides provide the advantages of biodegradability and safety and are thus expected to be widely employed as novel drug delivery vehicles. In this study, eutectogels were prepared using xanthan gum as the biodegradable gelator and choline chloride-xylitol as the model solvent since it has good solubility in quercetin. An MTT assay indicated that the eutectogel had excellent biocompatibility as its corresponding hydrogel. Meanwhile, water was found to play an important role in the preparation process and gel properties of xanthan gum-based eutectogels. Compared with its corresponding hydrogel, the xanthan gum-based eutectogel had better viscoelastic properties, higher thermal stability, and more defined shear thinning behavior; however, its structural recovery was relatively poor. Although eutectogels with different water contents displayed similar viscoelastic properties and thermal stability, gels with lower water contents were shown to have higher hardness and adhesiveness. According to a DSC analysis, the various rheological and texture properties of the eutectogels were attributed to changes in water states, which were influenced by the hydrogen bonding network of NADES. Owing to its degradability and biocompatibility, combined with the excellent solubility of NADES to bioactive ingredients, the novel eutectogel thus showed great potential for applications in fields, such as medicine and food.

## Figures and Tables

**Table 1:** Tan δ values for eutectogels with different water contents measured at 1 Hz of frequency.

**Water Content**	0%	20%	40%	60%	80%	100%
G′	0.21	674.91	776.53	667.23	639.71	375.42
G″	40.74	162.90	118.25	102.82	95.64	65.27
Tan δ	190.11	0.24	0.15	0.15	0.15	0.17

G′: Elastic modulus; G′′: Viscosity modulus; Tan δ: Loss tangent.

## Supplementary Materials

The following are available online, Figure S1: Solubility of quercetin in ChCl-Xyl with different water content at 25 °C. The solubility of quercetin measured in weight fraction, Wq of quercetin., Figure S2: Molecular structural formula of xanthan gum (**a**), choline chloride(**b**) and xylitol (**c**).
