# Supplementary material for: Novel Biocompatible Polysaccharide-Based Eutectogels with Tunable Rheological, Thermal, and Mechanical Properties: The Role of Water
Source: Molecules. 2020 Jul 22;25(15):3314. doi: 10.3390/molecules25153314 (PMC7435784; doi:10.3390/molecules25153314)
Supplement: Supplementary file 1 [file molecules-25-03314-s001.pdf]

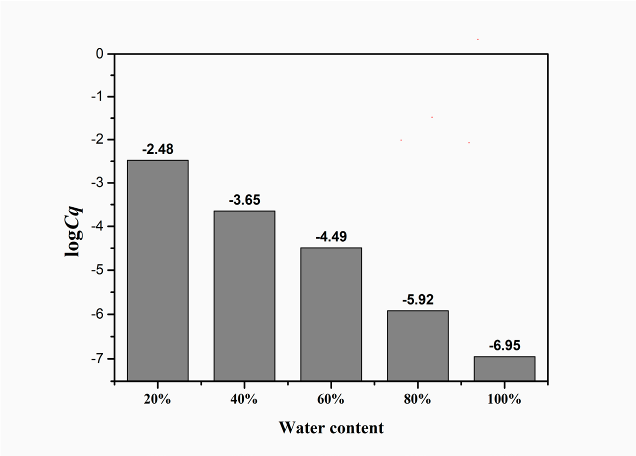

Water content in ChCl-Xyl (1:1)	20%	40%	60%	80%	100%
Quercetin solubility (mg/g)	2.13	0.22	3.25×10^{-2}	1.19×10^{-3}	1.12×10^{-4}

Figure S1. Solubility of quercetin in ChCl-Xyl with different water content at 25℃. The solubility of quercetin measured in weight fraction, Wq of quercetin.

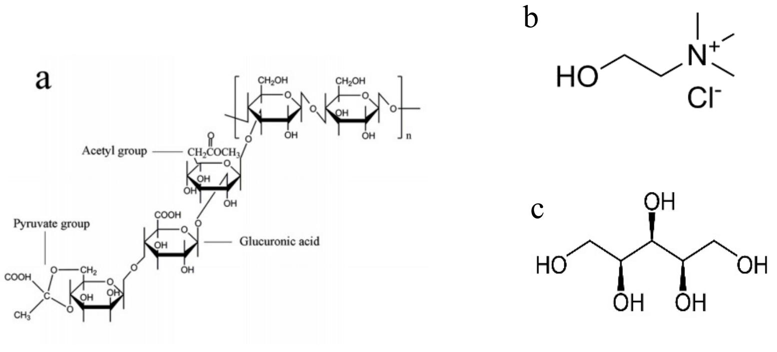

Figure.S2. Molecular structural formula of xanthan gum (a), choline chloride(b) and xylitol (c).
